# Slingshot homolog-1 mediates the secretion of small extracellular vesicles containing misfolded proteins by regulating autophagy cargo receptors and actin dynamics

**DOI:** 10.3389/fnagi.2022.933979

**Published:** 2022-08-25

**Authors:** Sara Cazzaro, Cenxiao Fang, Hirah Khan, Richard Witas, Teresa R. Kee, Jung-A. A. Woo, David E. Kang

**Affiliations:** ^1^Department of Pathology, School of Medicine, Case Western Reserve University, Cleveland, OH, United States; ^2^Department of Molecular Medicine, USF Health Morsani College of Medicine, Tampa, FL, United States; ^3^Louis Stokes Cleveland VA Medical Center, Cleveland, OH, United States

**Keywords:** SSH1, amyloid, tau, exosome, p62, optineurin, actin dynamics, autophagy

## Abstract

Increasing evidence indicates that the accumulation misfolded proteins in Alzheimer’s disease (AD) arises from clearance defects in the autophagy-lysosome pathway. Misfolded proteins such as Aβ and tau are secreted in small extracellular vesicles (i.e., exosomes) and are propagated from cell to cell in part through secreted small extracellular vesicles (sEVs). Recent studies suggest that autophagic activity and exosome secretion are coregulated events, and multiple autophagy-related proteins are found in sEVs, including the cargo receptors Sqstm1/p62 and optineurin. However, whether and how autophagy cargo receptors *per se* regulate the secretion of sEVs is unknown. Moreover, despite the prominent role of actin dynamics in secretory vesicle release, its role in EV secretion is unknown. In this study, we leveraged the dual axes of Slingshot Homolog-1 (SSH1), which inhibits Sqstm1/p62-mediated autophagy and activates cofilin-mediated actin dynamics, to study the regulation of sEV secretion. Here we show that cargo receptors Sqstm1/p62 and optineurin inhibit sEV secretion, an activity that requires their ability to bind ubiquitinated cargo. Conversely, SSH1 increases sEV secretion by dephosphorylating Sqstm1/p62 at pSer403, the phospho-residue that allows Sqstm1/p62 to bind ubiquitinated cargo. In addition, increasing actin dynamics through the SSH1-cofilin activation pathway also increases sEV secretion, which is mimicked by latrunculin B treatment. Finally, Aβ42 oligomers and mutant tau increase sEV secretion and are physically associated with secreted sEVs. These findings suggest that increasing cargo receptor engagement with autophagic cargo and reducing actin dynamics (i.e., SSH1 inhibition) represents an attractive strategy to promote misfolded protein degradation while reducing sEV-mediated cell to cell spread of pathology.

## Introduction

Emerging evidence indicates that the release and uptake of extracellular vesicles (EVs) represents an important form of intercellular communication that could transmit beneficial or pathogenic signals across different cells (Rajendran et al., 2014). Normal EVs have been shown to regulate neuronal neurite outgrowth, survival, and synaptic activity (Wang et al., 2011; Antonucci et al., 2012) as well as microglial activation (Verderio et al., 2012; Prada et al., 2013). EVs are secreted by all cell types and contain specific sets of RNA and proteins (membrane and cytosolic) (Valadi et al., 2007; Skog et al., 2008). Large microvesicles (MVs) (150–1000 nm) are derived from evagination and excision of the plasma membrane, whereas the smaller exosomes (30–150 nm) are intraluminal vesicles (ILVs) formed by inward budding of the limiting membrane of late endosomes or multivesicular bodies (MVBs), and are secreted by fusion with the plasma membrane (Raposo and Stoorvogel, 2013). Despite their ubiquitous presence in the brain, the role of EVs in brain cells and their potential effects on neurodegenerative processes are relatively unknown. In pathological settings, proteins prone to aggregation (i.e., Aβ, Tau, α-synuclein, and prion) are secreted in EVs, potentially providing an avenue for spreading such misfolded proteins to neighboring healthy cells (Rajendran et al., 2006; Vella et al., 2007; Danzer et al., 2012; Saman et al., 2012). For example, the spread of tau requires neighboring microglia and exosomes (Asai et al., 2015), and tau overexpression also abnormally recruits other proteins to exosomes (Saman et al., 2014). Alzheimer’s disease (AD) brain-derived exosomes contain tau and Aβ oligomers that can seed and propagate pathology (Sardar Sinha et al., 2018; Miyoshi et al., 2021). Other studies have shown beneficial effects of certain exosomes in reducing amyloid load and protecting against synaptic plasticity deficits (An et al., 2013; Yuyama et al., 2014).

Recent studies suggest that EV secretion of pathological proteins and autophagy pathways may be linked. For example, autophagy activation with the mTOR inhibitor rapamycin inhibits exosomal prion release, whereas inhibition of autophagy increases exosomal prion secretion (Abdulrahman et al., 2018). In a similar vein, inhibition of lysosome function by bafilomycin A1 increases TDP-43 and α-synuclein release in EVs (Iguchi et al., 2016; Minakaki et al., 2018), suggesting that failure of the autophagy-lysosome pathway (ALP) directs autophagosomes containing misfolded proteins for secretion in association with EVs. This secretory pathway has been proposed to involve the fusion of autophagosomes with MVBs to form amphisomes, which are then secreted by fusion with the plasma membrane (Gordon et al., 1992; Liou et al., 1997; Fader et al., 2008; Ganesan and Cai, 2021). Interestingly, autophagy cargo receptors, such as Sqstm1/p62 and optineurin, are often found in secreted exosomes (Gudbergsson and Johnsen, 2019), while they play vital roles in the clearance of misfolded proteins such as tau in AD (Xu et al., 2019; Woo et al., 2020; Fang et al., 2021; Roca-Agujetas et al., 2021a,b). However, whether prototypic autophagy cargo receptors regulate exosome secretion is unknown. Moreover, despite the crucial role of actin dynamics in secretory vesicle docking and secretion (Li et al., 2018), how actin dynamics regulate exosome secretion is also unknown. We and others previously showed that the protein phosphatase Slingshot Homolog-1 (SSH1) contains two major separable activities in the control of actin dynamics through cofilin activation through dephosphorylation at Ser3 (Niwa et al., 2002) and autophagy inhibition through p62 dephosphorylation at pSer403 (Fang et al., 2021). In this study, using a combination of DiI labeling of EVs and differential centrifugation, we explored the role of autophagy cargo receptors and actin dynamics regulated by SSH1 in small EV (sEV, aka exosome) secretion. Our findings highlight the roles of autophagy cargo receptors and actin dynamics in regulating sEV secretion through mechanisms impacting AD pathogenesis.

## Materials and methods

### Cell lines

Mouse hippocampus-derived neuroblastoma cells HT22, mouse embryonic fibroblast-derived NIH3T3, and tetracycline-inducible human embryonic kidney 293 cells overexpressing tau P301L (iHEK P301L) were cultured in Dulbecco’s modified Eagle’s medium (DMEM 1X) (Gibco, 11965-092) supplemented with 10% fetal bovine serum (FBS) (Sigma, 12306C) and 1% penicillin-streptomycin (P/S) (Gibco, 15140-122), and BM cyclin (Roche, 10799050001). iHEKP301L cells were induced with 1 μg/mL tetracycline when seeded, and cells were grown for 48 h before performing experimental assays. Cells were maintained at 37°C with 5% CO_2_ levels.

### Primary neurons

Cortical primary neurons were cultured from C57BL6 or tauP301S P0 mouse pups as previously described (Woo et al., 2015b; Fang et al., 2021). Briefly, the cortex was dissected in ice-cold HBSS and digested with 0.25% Trypsin-EDTA (1X) (Gibco, 25200-056), then plated in culture-treated plates with neurobasal medium (Invitrogen, 21103049) supplemented with 2% GlutaMAX (Invitrogen, 35050061) and 2% B27 supplement (Invitrogen, 17504044). Cells were maintained at 37°C with 5% CO_2_ levels.

### DNA constructs

pMXs-puro GFP-p62 (Addgene, 38277) (Itakura and Mizushima, 2011), pMXs-puro GFP-p62ΔC (addgene, 38282) (Itakura and Mizushima, 2011), ECFP-SSH1ΔC (Kurita et al., 2008), pEGFP-N1 human cofilin WT (addgene, 50859) (Garvalov et al., 2007), pEGFP-N1 human cofilin S3A (addgene, 50860) (Garvalov et al., 2007), pEGFP-N1 human cofilin S3E (addgene, 50861) (Garvalov et al., 2007), pOPTN-EGFP (addgene, 27052) (Park et al., 2006), pOPTN E478G-EGFP (addgene, 68848) (Turturro et al., 2014) were obtained from corresponding sources. Plasmids p3xFlag-SSH1 and p3xFlag-SSH1ΔN, and GFP-p62 403E were generated in the Kang lab as previously documented (Fang et al., 2021).

### DNA transfections

DNA plasmids were transiently transfected using Lipofectamine 2000 (Invitrogen, 11668-019) and reduced serum media Opti-MEM I (Gibco, 31985-070). After 4–6 h post-transfection, the media was replaced with new complete medium. Cells were grown for 48 h after transfection.

### Small extracellular vesicle (exosome) isolation

Small extracellular vesicles within the size range of exosomes were isolated by serial centrifugation as previously described (Witas et al., 2017). Briefly, 48 h after transfection, cells were washed twice with PBS (Gibco, 10010-023), and media was replaced with DMEM 1X supplemented with 10% exosome-depleted fetal bovine serum (FBS) (Gibco, A27208-03) and 1% P/S. Twenty-four hours later, media was collected for EV isolation. Samples were centrifuged at 4°C at 800 × *g* for 10 min to remove cells and large bodies, and supernatants were centrifuged at 4°C at 2,500 × *g* for 15 min to remove cell debris. Supernatants were then centrifuged at 4°C at 10,000 × *g* for 30 min to separate pellet microvesicles. Finally, the resulting supernatants were labeled with DiI and centrifuged 4°C at 100,000 × *g* for 1 h to pellet small EVs. Small EV pellets were washed with PBS and used for analysis.

### DiI staining

Vesicles were stained with DiI as previously described (Witas et al., 2017). Briefly, post-microvesicle supernatants were rocked for 15 min in 5 μM DiI (Thermo Fisher, D3911) solution at 37°C. After EVs isolation and washing, pellets were resuspended in 5 μl of fluorochrome mounting solution (Thermo scientific, TA-030-FM), and the entire volume was placed on a glass slide and covered with a glass coverslip for imaging and analysis.

### Nanoparticle tracking analysis

Isolated EVs were resuspended in 500 μl of PBS and ran on NanoSight™ LM10 with nanoparticle tracking analysis (Malvern). Using the NanoSight software, three videos of 60 s each were taken and analyzed for particle size distribution.

### Protein extraction

For cell protein extraction, cells were washed with 1× PBS and then resuspended in RIPA lysis buffer (50 mM Tris–HCl, pH 7.4, 150 mM NaCl, 2 mM EDTA, and 1% Triton X-100 [Amresco, 0694–1 L], 0.1% SDS) with protease inhibitor (GeneDEPOT, P3100-010) and phosphatase inhibitor (GeneDEPOT, P3200-005). Sample concentrations were determined and equalized using Pierce BCA Protein Assay Kit (Thermo Scientific, 23225), and then used for immunoblotting. For EV protein extraction, EVs isolated from the media of two 10 cm plates were resuspended in RIPA buffer with protease and phosphatase inhibitors. The samples were equalized based on corresponding protein concentrations in cell lysates.

### Western blotting

Equal protein amounts were loaded and ran in SDS-PAGE gels, then transferred to nitrocellulose membranes (GE Healthcare, 10600002). Blots were probed with primary antibodies overnight at 4°C (1:1000 dilution in TBS-T) and with HRP-conjugated secondary antibodies overnight at 4°C (1:1000 dilution in TBS-T) before detection with ECL western blot reagents (Pierce, 34578) and imaged with Fuji LAS-4000 imager (LAS-4000, Pittsburgh, PA, United States). All images were quantified using NIH ImageJ software.

### Antibodies

Antibodies were purchased from the following commercial sources: Aβ (Cell Signaling Technology, 8243S), Tau (Santa Cruz Biotech, sc-390476), Hsc70 (Enzo Life Sciences, ADI-SPA-815-F), TSG101 (Thermo Fisher, PA5-31260), GFP (Cell Signaling Technology, 2956S), β-Actin (Santa Cruz Biotech, sc-47778), p62 (Cell Signaling Technology, 5114), Flag (Sigma Aldrich, F1804), SSH1 (ECM Biosciences, SP1711) (Cell Signaling Technology, 13578), Cofilin (Cell Signaling Technology, 5175s), Peroxidase-Conjugated AffiniPure Goat anti-mouse IgG (Jackson ImmunoResearch, 115-035-033), Peroxidase-conjugated AffiniPure Goat anti-rabbit IgG (Jackson ImmunoResearch, 111-035-033), donkey anti-rat IgG-HRP (Southern Biotech, 6430-05).

### Drugs, reagents, and oligonucleotides

Amyloid-β 1-42 (GenicBio, A-42-T-1), Rapamycin (Sigma Aldrich, R0395), Bafilomycin A1 (Sigma Aldrich, B1793), Latrunculin B (Sigma Aldrich, L5288), Jasplakinolide (AdipoGen Life Sciences, 102396-24-7), p62 siRNA (Cell Signaling Technology, 6399S), SSH1 siRNA (Dharmacon GE Healthcare, 5′-GAG GAG CUG UCC CGA UGA C-3′), Cofilin siRNA (Dharmacon GE Healthcare, 5′-GGA GGA CCU GGU GUU CAU C-3′).

### Imaging and quantification

Confocal images were captured with the Olympus FV10i confocal microscope (Tokyo, Japan). All images were quantified using NIH ImageJ software.

## Results

### Rapamycin decreases and bafilomycin increases small extracellular vesicle secretion

**FIGURE 1:**
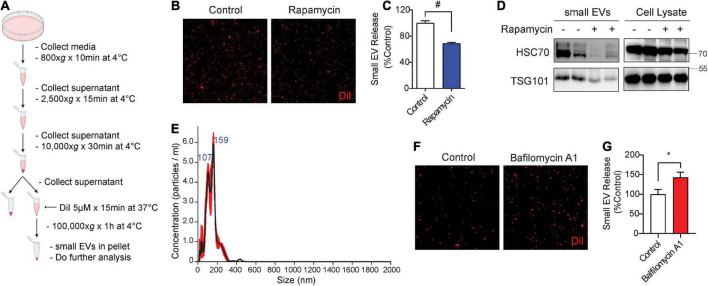
Rapamycin decreases and bafilomycin A1 increases small EV secretion. **(A)** Schematic of small EV isolation method. **(B)** Representative images of DiI-stained small EVs secreted from NIH3T3 cells with or without 200 nM rapamycin treatment for 24 h. **(C)** Quantification of secreted small EVs for **(B)**. Data are presented as means ± SEM. *n* = 3 independent experiments, *t*-test, ^#^*P* < 0.0001. **(D)** Representative immunoblots for the indicated proteins in secreted small EVs and cell lysates from NIH3T3 cells with or without 200 nM rapamycin treatment for 24 h. **(E)** Representative NTA analysis of small EVs isolated from NIH3T3 cells. The black line indicates concentration and size of particles, and the red line indicates standard error. **(F)** Representative images of DiI-stained small EVs secreted from NIH3T3 cells with or without 200 nM bafilomycin A1 treatment for 6 h. **(G)** Quantification of secreted small EVs for **(F)**. Data are presented as means ± SEM. *n* = 4 independent experiments, *t*-test, *P = 0.02.

To measure the secretion of exosomes, hereafter referred to as small EVs (sEVs), we used medium containing exosome-depleted (exo-free) FBS to collect conditioned medium from cells. Post-microvesicle (10,000 × *g*) supernatant was labeled with the fluorescent lipophilic dye DiI and ultracentrifuged at 100,000 × *g* to pellet sEVs (Figure 1A). We then visualized and quantified isolated sEVs on microscopic slides by fluorescence confocal microscopy. A similar method of DiI-labeling of EVs has previously been documented (Lehmann et al., 2008; Beer et al., 2015; Witas et al., 2017). As earlier studies showed that the autophagy activator and mTOR inhibitor rapamycin significantly inhibits sEV secretion (Fader et al., 2008; Abdulrahman et al., 2018; Bhat et al., 2021), we first tested the effects of rapamycin on sEV secretion in NIH3T3 cells. Indeed, rapamycin treatment for 24 h significantly reduced sEV secretion by ∼30% (Figures 1B,C). Western blotting for exosome markers Hsc70 and TSG101 showed corresponding reductions of both proteins in isolated sEVs but not in whole-cell lysates (Figure 1D). Nanoparticle tracking analysis (NTA) showed that the isolated sEVs are within the expected size range, the vast majority of which were under ∼150 nm in diameter with a minor proportion in the 150–250 nm range (Figure 1E). By contrast, inhibition of lysosomes with bafilomycin A1 treatment for 6 h significantly increased sEV release into the conditioned medium by ∼45% (Figures 1F,G), in agreement with previous studies (Iguchi et al., 2016; Minakaki et al., 2018).

### Autophagy cargo receptors, Sqstm1/p62 and optineurin, suppress secreted small extracellular vesicle secretion through the ubiquitin association domain

**FIGURE 2:**
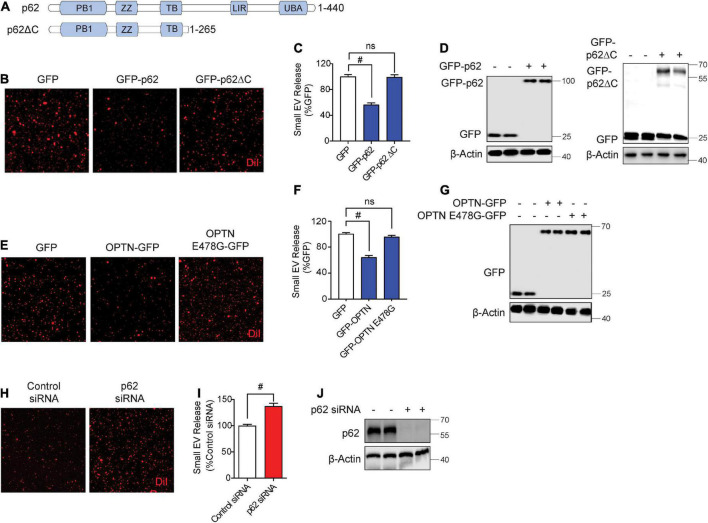
Autophagy cargo receptors, p62 and optineurin, inhibit small EV secretion through the ubiquitin association domain. **(A)** Schematic of p62 full length and p62ΔC proteins with their respective domains. **(B)** Representative images of DiI-stained small EVs secreted from NIH3T3 cells expressing GFP, GFP-p62, or GFP-p62ΔC. **(C)** Quantification of secreted small EVs for **(B)**. Data are presented as means ± SEM. *n* = 3 independent experiments, 1-way ANOVA, followed by Dunnett’s *post-hoc*, ^#^*P* < 0.0001. **(D)** Representative immunoblots of GFP-p62, GFP-p62ΔC, GFP, and β-Actin protein levels in lysates from cells for **(B)**. **(E)** Representative images of DiI-stained small EVs secreted from NIH3T3 cells expressing GFP, GFP-OPTN, or GFP-OPTN E478G. **(F)** Quantification of secreted small EVs for **(E)**. Data are presented as means ± SEM. *n* = 3 independent experiments, 1-way ANOVA, followed by Dunnett’s *post-hoc*, ^#^*P* < 0.0001. n.s. = not significant. **(G)** Representative immunoblots for GFP, GFP-OPTN, and β-Actin protein levels in cell lysates for **(E)**. **(H)** Representative images of DiI-stained small EVs secreted from NIH3T3 cells transfected with control siRNA or p62 siRNA. **(I)** Quantification of secreted small EVs for **(H)**. Data are presented as means ± SEM. *n* = 4 independent experiments, *t*-test, ^#^*P* < 0.0001. **(J)** Representative immunoblots for p62 and β-Actin protein levels in cell lysates for **(H)**.

While previous studies have observed autophagy cargo receptors such as Sqstm1/p62 and optineurin in isolated sEVs (Gudbergsson and Johnsen, 2019), whether such cargo receptors regulate sEV secretion is unknown. Hence, we tested if overexpression of Sqstm1/p62 or optineurin alters sEV secretion. Hereafter, we use p62 in reference to Sqstm1/p62. The C-terminal region of p62 contains the UBA and LIR domains essential for binding to ubiquitinated cargo and LC3, respectively (Figure 2A; Katsuragi et al., 2015). The UBA region of p62 is subject to phosphorylation at Ser403 by ULK1, CK2, and/or TBK1, allowing p62 to bind ubiquitinated cargo (Pilli et al., 2012; Katsuragi et al., 2015; Lim et al., 2015; Matsumoto et al., 2015; Sanchez-Martin and Komatsu, 2018). GFP-p62 transfection significantly decreased sEV secretion by ∼50% compared to control GFP transfection (Figures 2B–D). However, transfection of p62 lacking the UBA and LIR domains (p62ΔC) failed to alter sEV secretion (Figures 2B–D), suggesting that p62 engagement with ubiquitinated cargo and/or LC3 binding are necessary to inhibit sEV release. Like p62, transfection of optineurin also suppressed sEV secretion. However, the ALS-linked optineurin E478G mutant, which fails to bind ubiquitin (Nakazawa et al., 2016), also failed to alter sEV secretion (Figures 2E–G), indicating that ubiquitin binding is a necessary step in suppressing sEV secretion by optineurin. To determine if endogenous p62 normally inhibits sEV secretion, we transfected cells with p62 siRNA. As expected, p62 knockdown significantly increased sEV secretion by ∼40% (Figures 2H–J). Hence, these observations indicate that autophagy cargo receptors, p62 and optineurin, inhibit sEV secretion by engaging ubiquitinated cargo.

### Slingshot homolog-1-mediated p62 inhibition at pSer403 increases secreted small extracellular vesicle secretion

**FIGURE 3:**
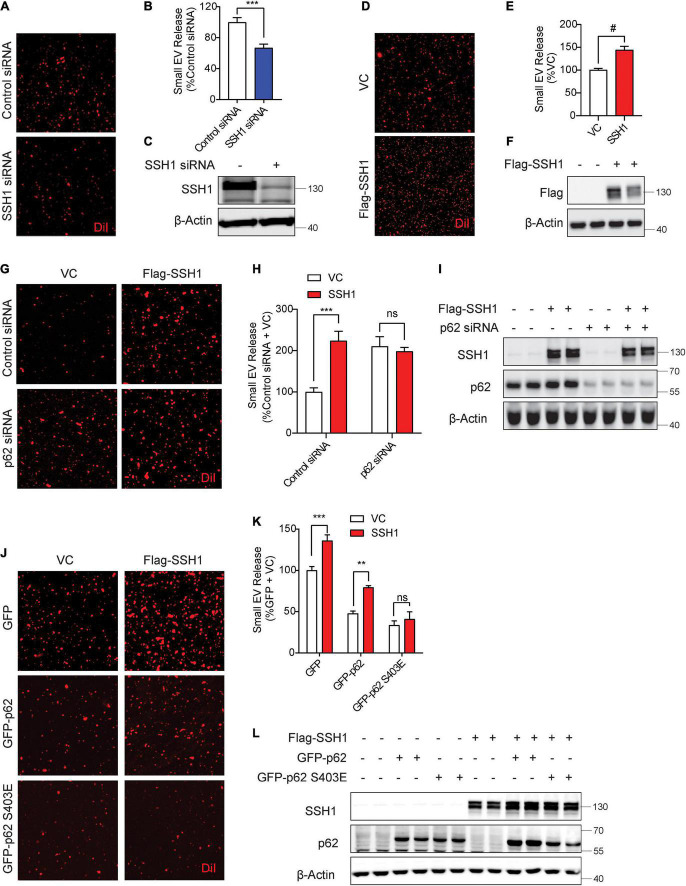
Slingshot homolog-1 (SSH1)-mediated p62 inhibition at pSer403 increases small EV secretion. **(A)** Representative images of DiI-stained small EVs secreted from NIH3T3 cells transfected with control siRNA or SSH1 siRNA. **(B)** Quantification of secreted small EVs for **(A)**. Data are presented as means ± SEM. *n* = 3 independent experiments, *t*-test, ***P = 0.0005. **(C)** Representative immunoblots for SSH1 and β-Actin protein levels in cell lysates for **(A)**. **(D)** Representative images of DiI-stained small EVs secreted from NIH3T3 cells expressing VC or Flag-SSH1 **(E)** Quantification of secreted small EVs for **(D)**. Data are presented as means ± SEM. *n* = 3 independent experiments, *t*-test, ^#^*P* < 0.0001 **(F)** Representative immunoblots for Flag and β-Actin protein levels in cell lysates for **(D)**. **(G)** Representative images of DiI-stained small EVs secreted from NIH3T3 cells co-transfected with VC or Flag-SSH1 and control siRNA or p62 siRNA. **(H)** Quantification of secreted small EVs for **(G)**. Data are presented as means ± SEM. *n* = 3 independent experiments, 2-way ANOVA, followed by Sidak’s *post-hoc*, ***P = 0.0004. n.s. = not significant. **(I)** Representative immunoblots showing SSH1, p62, and β-Actin protein levels in cell lysates for **(G)**. **(J)** Representative images of DiI-stained small EVs secreted from NIH3T3 cells co-transfected with VC or Flag-SSH1 and GFP, GFP-p62, or GFP-p62 S403E. **(K)** Quantification of secreted small EVs for **(J)**. Data are presented as means ± SEM. *n* = 3 independent experiments, 2-way ANOVA, followed by Sidak’s *post-hoc*, ***P = 0.0007, **P = 0.001. n.s. = not significant. **(L)** Representative immunoblots for SSH1, p62, and β-Actin protein levels in cell lysates for **(J)**.

We recently showed that SSH1 contains a modular activity in inhibiting p62 autophagy flux by dephosphorylating p62 at pSer403, the phospho-residue that allows p62 binding to ubiquitinated cargo (Fang et al., 2021). SSH1 siRNA significantly decreased sEV secretion by nearly ∼40% (Figures 3A–C), indicating that endogenous SSH1 promotes sEV secretion. Conversely, forced expression of SSH1 significantly increased sEV secretion (Figures 3D–F). In p62 depleted cells, which exhibited significantly elevated sEV secretion (Figures 3G–I), SSH1 failed to increase sEV secretion (Figures 3G–I), indicating that SSH1-induced sEV secretion requires p62. To determine if SSH1-induced sEV secretion is specifically through p62 dephosphorylation at pSer403, we co-transfected cells with or without SSH1 and GFP control, GFP-p62, or GFP-p62-S403E, the latter mutant which mimics constitutive p62 phosphorylation at Ser403 (Fang et al., 2021). As expected, SSH1 overexpression significantly increased sEV secretion in the setting of GFP transfection and significantly reversed the decline in sEV secretion induced by GFP-p62 (Figures 3J–L). However, SSH1 failed to change the reduction in sEV secretion caused by GFP-p62-S403E (Figures 3J–L), indicating that SSH1 increases sEV secretion by dephosphorylating p62 at pSer403, a step that renders p62 inactive for binding to ubiquitinated cargo. Hence, these results indicate that the ability of p62 to engage ubiquitinated cargo is critical to SSH1-regulated sEV secretion. We did not investigate SSH1-mediated effects on optineurin-induced inhibition of sEV secretion, as SSH1 does not alter optineurin-mediated autophagy (Fang et al., 2021).

### Slingshot homolog-1 increases secreted small extracellular vesicle secretion partially through cofilin activation and F-actin disruption

**FIGURE 4:**
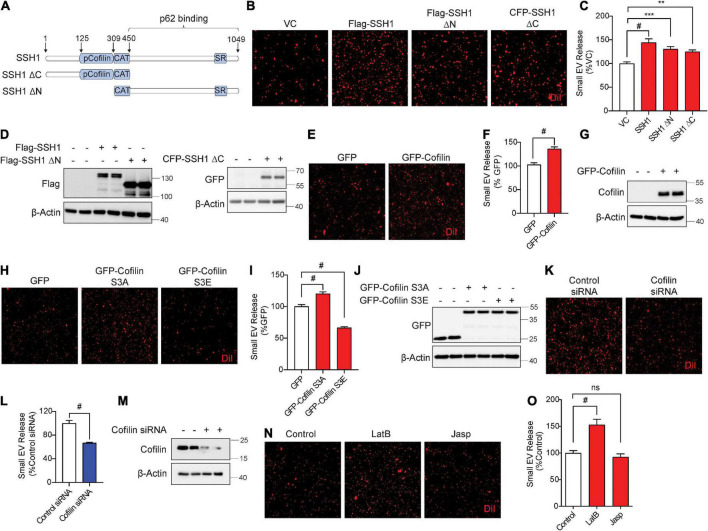
Slingshot homolog-1 (SSH1) increases small EV secretion partially through cofilin activation and F-actin disruption. **(A)** Schematic of SSH1 full length, SSH1ΔC, and SSH1ΔN proteins. **(B)** Representative images of DiI-stained small EVs secreted from NIH3T3 cells expressing VC, Flag-SSH1, Flag-SSH1ΔN, or CFP-SSH1ΔC. **(C)** Quantification of secreted small EVs for **(B)**. Data are presented as means ± SEM. *n* = 3 independent experiments, 1-way ANOVA, followed by Dunnett’s *post-hoc*, ^#^*P* < 0.0001, ***P = 0.001, **P = 0.0069. **(D)** Representative immunoblots for Flag, GFP, and β-Actin protein levels in cell lysates for **(B)**. **(E)** Representative images of DiI-stained small EVs secreted from NIH3T3 cells expressing GFP or GFP-Cofilin. **(F)** Quantification of secreted small EVs for **(E)**. Data are presented as means ± SEM. *n* = 3 independent experiments, *t*-test, ^#^*P* < 0.0001. **(G)** Representative immunoblots for cofilin and β-Actin protein levels in cell lysates for **(E)**. **(H)** Representative images of DiI-stained small EVs secreted from NIH3T3 cells expressing GFP, GFP-cofilin-S3A, or GFP-cofilin-S3E. **(I)** Quantification of secreted small EVs for **(H)**. Data are presented as means ± SEM. *n* = 3 independent experiments, 1-way ANOVA, followed by Dunnett’s *post-hoc*, ^#^*P* < 0.0001. **(J)** Representative immunoblots for GFP and β-Actin protein levels in cell lysates for **(H)**. **(K)** Representative images of DiI-stained small EVs secreted from NIH3T3 cells transfected with control siRNA or cofilin siRNA. **(L)** Quantification of secreted small EVs for **(K)**. Data are presented as means ± SEM. *n* = 3 independent experiments, *t*-test, ^#^*P* < 0.0001. **(M)** Representative immunoblots for cofilin and β-Actin protein levels in cell lysates for **(K)**. **(N)** Representative images of DiI-stained small EVs secreted from NIH3T3 cells with or without 100 nM Latrunculin B (LatB) or 100 nM Jasplakinolide (Jasp) treatment for 8 h. **(O)** Quantification of secreted small EVs for **(N)**. Data are presented as means ± SEM. *n* = 3 independent experiments, 1-way ANOVA, followed by Dunnett’s *post-hoc*, ^#^*P* < 0.0001. n.s. = not significant.

Slingshot homolog-1 was initially discovered as the major phosphatase that activates the actin severing and depolymerizing protein cofilin by its dephosphorylation at pSer3, increasing actin dynamics (Niwa et al., 2002; Kurita et al., 2007, 2008). SSH1-mediated dephosphorylation of cofilin and p62 are separable activities, as SSH1ΔN lacking the cofilin binding site dephosphorylates p62 but not cofilin, while the SSH1ΔC lacking the p62 binding site dephosphorylates cofilin but not p62 (Fang et al., 2021; Figure 4A). Hence, we tested if SSH1ΔN and SSH1ΔC increase sEV secretion compared to vector control and full-length SSH1. Surprisingly, SSH1ΔN or SSH1ΔC overexpression significantly increased sEV secretion compared to vector control, albeit less effectively than full-length SSH1 (Figures 4B–D). Given that SSH1ΔC increases cofilin activation, we next tested whether cofilin *per se* increases sEV secretion. Indeed, cofilin overexpression alone significantly increased sEV secretion by ∼30% (Figures 4E–G). The constitutively active cofilin-S3A similarly increased sEV secretion, whereas the inactive and dominant-negative cofilin-S3E (Liu et al., 2017; Shaw and Bamburg, 2017; Woo et al., 2019), which mimics the phosphorylated state, significantly decreased sEV secretion by ∼40% (Figures 4H–J). Moreover, cofilin siRNA significantly reduced sEV secretion by ∼35% (Figures 4K–M), indicating that endogenous cofilin promotes sEV secretion.

As actin filament severing and depolymerization activity of cofilin promoted sEV secretion, we tested if latrunculin B and jasplakinolide, drugs known to inhibit actin polymerization (Wakatsuki et al., 2001) and nucleate actin (Bubb et al., 2000), respectively, alter sEV secretion. Compared to the vehicle control, preventing actin polymerization by latrunculin B significantly increased sEV secretion by ∼50%, whereas promoting actin nucleation by jasplakinolide had no significant effect on sEV secretion (Figures 4N,O). These results are consistent with the observation that activated cofilin, which severs and depolymerizes F-actin, increases sEV release.

### Aβ42 oligomers and misfolded tau increase secreted small extracellular vesicle secretion and are associated with secreted small extracellular vesicles

**FIGURE 5:**
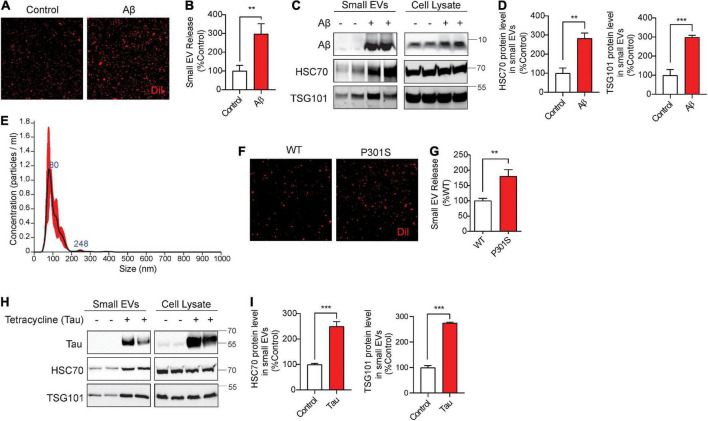
Aβ42 oligomers (Aβ42) oligomers and misfolded tau increase small EV secretion and are associated with secreted small EVs. **(A)** Representative images of DiI-stained small EVs secreted from HT22 cells with or without 250 nM Aβ treatment for 24 h. **(B)** Quantification of secreted small EVs for **(A)**. Data are presented as means ± SEM. *n* = 3 independent experiments, *t*-test, **P < 0.005. **(C)** Representative immunoblots for the indicated proteins in secreted small EVs and cell lysates from HT22 cells with or without 250 nM Aβ treatment for 24 h. **(D)** Quantification of Hsc70 and TSG101 (small EV markers) for **(C)**. Data are presented as means ± SEM. *n* = 4 independent experiments, *t*-test, ***P = 0.0007, **P < 0.004. **(E)** Representative NTA analysis of vesicle size distribution of small EVs isolated from HT22 cells. The black line indicates concentration and size of particles, and the red line indicates standard error. **(F)** Representative images of DiI-stained small EVs secreted by DIV10 cortical primary neurons derived from C57BL6 (WT) or tau^*P*301*S*^ (P301S) mice. **(G)** Quantification of secreted small EVs for **(F)**. Data are presented as means ± SEM. *n* = 3 independent experiments, *t*-test, **P = 0.002. **(H)** Representative immunoblots for the indicated proteins in secreted small EVs and cell lysates from tet-inducible iHEK-P301L cells expressing tau^*P*301*L*^, with or without tetracycline. **(I)** Quantification of Hsc70 and TSG101 (small EV markers) for **(H)**. Data are presented as means ± SEM. *n* = 4 independent experiments, *t*-test, ***P < 0.0003.

Aβ42 oligomers (Aβ42o) increase cofilin activation *via* SSH1, disrupt F-actin (Woo et al., 2015b) and enhance mTOR signaling (Caccamo et al., 2010). Moreover, misfolded mutant tau inhibits p62-mediated autophagy flux (Fang et al., 2021) and promotes F-actin bundling (Fulga et al., 2007; Cabrales Fontela et al., 2017). We tested if these AD signature pathologies alter sEV secretion and are in turn associated with sEVs. For treatment with Aβ42o, we used mouse hippocampus-derived HT22 neuroblastoma cells, as HT22 cells are responsive to Aβ42o-induced cofilin activation (Woo et al., 2015b). Aβ42o were prepared as previously described (Woo et al., 2015b). Aβ42o (250 nM) treatment to HT22 cells for 24 h significantly increased sEV secretion by nearly ∼3-fold (Figures 5A,B). Likewise, Western blotting for Hsc70 and TSG101 also demonstrated significant increases in these exosome markers by Aβ42o treatment in isolated sEVs but not in cell lysates (Figures 5C,D). The treated Aβ42o were readily detected in isolated sEVs (Figure 5C). Like in NIH3T3 cells, isolation of DiI-labeled sEVs in HT22 cells yielded the vast majority of nanovesicles <150 nm in diameter by NTA (Figure 5E), consistent with the range for exosomes. To determine whether misfolded tau alters sEV secretion, we utilized Frontotemporal dementia with Parkinsonism-17 (FTDP-17) tau^*P*301*S*^ mutant and littermate WT neurons from P0 pups grown for 9 days *in vitro* (DIV9). On DIV9, the media was replaced with fresh media and collected after 24 h. Tau^*P*301*S*^ neurons exhibited significantly increased sEV secretion compared to littermate WT neurons (Figures 5F,G). To confirm this finding in a different way using another FTDP-17-linked tau mutation, we utilized tetracycline-inducible HEK293 expressing the tau^*P*301*L*^ mutation. Tetracycline-inducible expression of tau^*P*301*L*^ significantly increased exosome markers Hsc70 and TSG101 in isolated sEVs but not in cell lysates (Figures 5H,I), indicating that misfolded tau increases sEV secretion in neurons and HEK293 cells. Tau^*P*301*L*^ was also readily detected in association with isolated sEVs (Figure 5H), indicating that misfolded tau not only drives sEV release but is also a content of sEVs.

## Discussion

Multiple studies have implicated the involvement of the SSH1-cofilin pathway in Aβ-induced dendritic spine shrinkage and F-actin loss (Zhao et al., 2006; Shankar et al., 2007) as well as the accumulation of multiple pathologies, including cofilin-actin pathology (Minamide et al., 2000; Rahman et al., 2014), Aβ pathology (Liu et al., 2019), and tau pathology (Woo et al., 2019). Cofilin activity is increased in the brains of AD patients (Zhao et al., 2006; Kim et al., 2013) and the APP/PS1 mouse model (Woo et al., 2015b). At the same time, the autophagy-lysosome pathway is overwhelmed and impaired in AD (Lin et al., 2003; Nixon et al., 2005; Salminen et al., 2012; Hebron et al., 2014; Rea et al., 2014; Tanji et al., 2014; Feng et al., 2019; Roca-Agujetas et al., 2021a,b), which contributes to the accumulation of misfolded proteins and dysfunctional mitochondria (Ramesh Babu et al., 2008; Xu et al., 2019; Woo et al., 2020; Fang et al., 2021; Roca-Agujetas et al., 2021a,b). Meanwhile, AD pathological proteins Aβ and tau are secreted in exosomes and are propagated from cell to cell in part *via* exosomes (Asai et al., 2015; Sardar Sinha et al., 2018; Miyoshi et al., 2021). Yet, the pathobiological relationship between these drivers of AD pathogenesis and sEV secretion are unknown. In this study, we took advantage of the dual axes of SSH1 signaling in the inhibition of autophagy (Fang et al., 2021) and the promotion of actin dynamics (Niwa et al., 2002; Kurita et al., 2007, 2008) to study the corresponding pathways in sEV secretion. We used the neutral term sEVs to place no bias on the origin of the EVs; however, sEVs are largely equivalent to exosomes based on their predominant size range. Here we showed for the first time that autophagy cargo receptors, p62 and optineurin, inhibit sEV secretion, an activity that requires their ability to engage ubiquitinated cargo. SSH1, which inhibits p62 binding to cargo (Fang et al., 2021), increased sEV secretion, but not in p62-depleted cells or cells expressing the p62-S403E mutation. However, the N-terminal domain of SSH1 (SSH1ΔC), which activates cofilin but does not inhibit p62 (Fang et al., 2021), increased sEV secretion through the cofilin activation pathway. Accordingly, latrunculin B, which prevents actin polymerization and indirectly promotes F-actin depolymerization (Wakatsuki et al., 2001), mimicked the effect of cofilin in enhancing sEV secretion. Finally, we showed that AD pathological proteins, Aβ oligomers and mutant tau, both of which impact autophagy and F-actin dynamics pathways (Fulga et al., 2007; Caccamo et al., 2010; Woo et al., 2015b; Cabrales Fontela et al., 2017; Fang et al., 2021), increased sEV secretion and are also contents of secreted sEVs. These findings, therefore, implicate autophagy cargo receptor-cargo interactions and actin dynamics in regulating sEV secretion through mechanisms contributing to AD pathogenesis.

**FIGURE 6:**
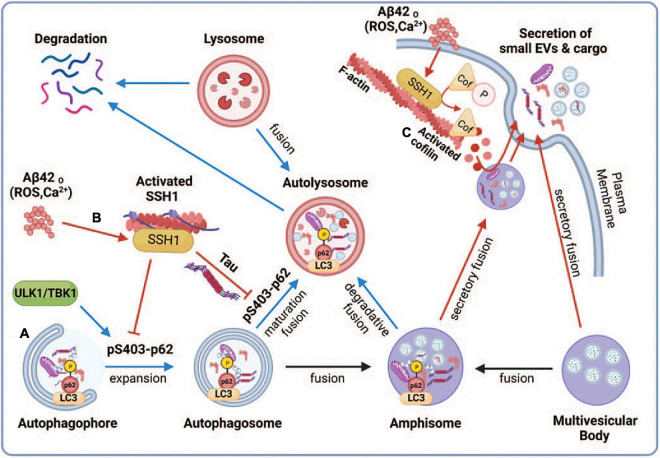
Proposed model depicting the regulation of small EV secretion by p62-mediated autophagy and cofilin-mediated actin dynamics through the SSH1 pathway. Note that blue lines/arrows favor lysosomal degradation, whereas red lines/arrows favor secretory clearance. **(A)** Accumulation of misfolded proteins or dysfunctional mitochondria activates autophagy through ULK1 and TBK1, which phosphorylate p62 at Ser403, resulting in p62 binding to ubiquitinated cargo and activation of LC3 to drive autophagosome maturation. This promotes autophagy flux and fusion of autophagosomes and amphisomes with lysosomes, resulting in the degradation of ubiquitinated cargo together with intraluminal vesicles from amphisomes, thereby reducing small EV secretion. **(B)** Oxidative stress (ROS) or calcium elevation induced by Aβ oligomers or otherwise activates SSH1, which dephosphorylates p62 at pSer403 and inhibits p62 autophagy flux. Dephosphorylated p62 is less able to bind ubiquitinated cargo and activate LC3, which slows autophagy flux and fusion of autophagosomes and amphisomes with lysosomes. This process diverts amphisomes toward the secretory fusion pathway and increases small EV secretion. Like SSH1, inhibition of p62 autophagy flux by misfolded tau (Fang et al., 2021) also likely contributes to increased small EV secretion. **(C)** Actin filaments serve to recruit vesicles and create a diffusion barrier for vesicles to gain access to the plasma membrane. SSH1 activation by ROS or calcium also activates cofilin, which severs and depolymerizes the F-actin network near the plasma membrane. This process allows large secretory amphisomes to access membrane docking sites, facilitating small EV secretion. This illustration was generated at Biorender.com.

Multiple studies have shown that autophagosomes accumulate in AD brains (Lin et al., 2003; Nixon et al., 2005; Hebron et al., 2014; Feng et al., 2019), indicative of impairment in the autophagy-lysosome pathway. Autophagosomes can fuse with multivesicular bodies (MVBs) containing intraluminal vesicles to form amphisomes or fuse directly with lysosomes for degradation (Ganesan and Cai, 2021; Figure 6). Our observations that the ability of p62 and optineurin to inhibit sEV secretion depends on their ability to engage ubiquitinated cargo are intriguing in light of the significantly reduced p62 Ser403 phosphorylation in AD brains (Tanji et al., 2014), a phospho-residue required for p62 to bind ubiquitinated cargo (Pilli et al., 2012; Katsuragi et al., 2015; Lim et al., 2015; Matsumoto et al., 2015; Sanchez-Martin and Komatsu, 2018). We interpret this to indicate that the binding of autophagy cargo receptors to ubiquitinated cargo promotes autophagy flux and fusion of autophagosomes and amphisomes with lysosomes (Fang et al., 2021), which diverts amphisomes away from fusing with the plasma membrane and are instead degraded by lysosomes (Figure 6A). Indeed, cargo binding to p62 promotes autophagosome maturation by enhancing the synthesis of LC3 and its conversion to LC3-II (Cha-Molstad et al., 2017; Figure 6A). In support of this notion, SSH1 increased sEV secretion through its ability to dephosphorylate p62 at pSer403, which reduces cargo binding and inhibits p62 autophagy flux (Fang et al., 2021; Figure 6B). Interestingly, calcium elevation or reactive oxygen species (ROS) induced by Aβ oligomers or other stressors activate SSH1 (Davis et al., 2011; Woo et al., 2015a,b) through calcineurin activation (Wang et al., 2005; Tu et al., 2014) and/or 14-3-3 oxidation (Wang et al., 2005), respectively, the latter which releases SSH1 from 14-3-3 inhibitory control (Figure 6B). Such calcium and reactive oxygen species (ROS)-mediated control of SSH1 activation is consistent with our observation that Aβ42 oligomers also increase sEV secretion (Figure 6B). Like SSH1, inhibition of p62-mediated autophagy flux by misfolded tau (Fang et al., 2021) is also consistent with increased sEV secretion by mutant tau in this study, likely by diverting autophagosomes away from lysosomes toward secretory amphisomes (Figure 6B).

Control of actin dynamics is critical to maintaining cell morphology and other cellular processes, including membrane protein trafficking, endocytosis, phagocytosis, and exocytosis (Carlsson, 2018; Li et al., 2018; Liu et al., 2019). However, the role of actin dynamics in sEV secretion is essentially unknown. The actin-binding protein cortactin positively regulates sEV secretion by binding to F-actin and Arp2/3, stabilizing docking sites at the plasma membrane (Sinha et al., 2016). Like exocytosis of secretory vesicles, sEV secretion is increased by calcium elevation (Savina et al., 2003). At the microscale level, the actin-based cytoskeleton serves to both recruit vesicles and function as a diffusion barrier that prevents vesicles from gaining access to docking sites on the plasma membrane (Li et al., 2018). Hence, a dense network of actin filaments would serve as a far more significant barrier to the much larger MVBs or amphisomes. As SSH1-mediated cofilin activation is increased by calcium elevation (Wang et al., 2005), the F-actin severing and depolymerizing cofilin activities would be expected to trim the dense F-actin network, allowing MVBs and amphisomes to gain access to plasma membrane docking sites (Figure 6C). However, excessive depolymerization of F-actin could also hinder sEV secretion by failing to recruit MVBs or amphisomes to the F-actin mesh close to the plasma membrane. Studies on secretory vesicles have shown that it is the oscillation of calcium signals and actin dynamics (depolymerization and polymerization) that coordinately control vesicle access to docking sites and exocytosis (Li et al., 2018). Indeed, cofilin-S3A, which is far more active than wild-type (WT) cofilin, was slightly less effective than WT cofilin in increasing sEV secretion, which may be due to the excessive actin disruptive activity of this mutant. On the other hand, the inactive and dominant-negative cofilin-S3E significantly decreased sEV secretion, likely due to the inhibition of endogenous SSH1 (Liu et al., 2017; Shaw and Bamburg, 2017; Woo et al., 2019). Like cofilin, 100 nM latrunculin B treatment for 8 h, a dose that partially disrupts F-actin (Wakatsuki et al., 2001), had a net positive effect on sEV secretion.

Our studies showed that Aβ oligomers and misfolded tau not only increase sEV secretion but are also secreted in association with sEVs. This coupling appears to be a way for cells to efficiently expel misfolded proteins, which otherwise would impede and congest the autophagy machinery. Excessive secretion of misfolded proteins by “secretory autophagy” in association with sEVs or otherwise could then serve as seeds for the propagation of pathology from cell to cell (Rajendran et al., 2006; Vella et al., 2007; Danzer et al., 2012; Saman et al., 2012, 2014; Asai et al., 2015; Sardar Sinha et al., 2018; Miyoshi et al., 2021).

## Conclusion

Our data suggest that increasing cargo receptor engagement with autophagic cargo and reducing actin dynamics (i.e., SSH1 inhibition) represents an attractive strategy to promote misfolded protein degradation while reducing sEV-mediated cell to cell spread of pathology.

## Data availability statement

The original contributions presented in this study are included in the article/supplementary material, further inquiries can be directed to the corresponding authors.

## Ethics statement

The animal study was reviewed and approved by the Institutional Animal Care and Use Committee (IACUC) at the University of South Florida (USF) and Case Western Reserve University (CWRU).

## Author contributions

SC: investigation, methodology, and writing. CF, HK, and RW: investigation and methodology. TK: data analysis and visualization. JW: investigation, writing, review, and funding acquisition. DK: conceptualization, writing, visualization, and funding acquisition. All authors have read and approved the manuscript.

## Abbreviations

Aβ42o: Aβ42 oligomers
Arp2/3: actin related protein 2/3 complex
AD: Alzheimer’s disease
ALS: amyotrophic lateral sclerosis
ALP: autophagy-lysosome pathway
CK2: casein kinase 2
EVs: extracellular vesicles
sEVs: small EVs
F-actin: fIlamentous actin
FTDP-17: frontotemporal dementia with parkinsonism-17
Hsc70: heat shock cognate 70 kDa protein
FP: green fluorescent protein
ILVs: intraluminal vesicles
LIR: LC3 interacting region
LC3: microtubule-associated protein light chain 3
MVs: microvesicles
MVBs: multivesicular bodies
mTOR: mammalian target of rapamycin
NTA: nanoparticle tracking analysis
SSH1: slingshot homolog-1
sequestosome-1: Sqstm1/p62
TBK1: tank binding kinase 1
TSG101: tumor susceptibility gene 101 protein
UBA: ubiquitin association
ULK1: Unc-51 like autophagy activating kinase 1.
